# Solitary vertebral metastasis of unknown primary renal cell carcinoma treated with surgical resection plus tyrosine kinase inhibitor: A case report

**DOI:** 10.1016/j.ijscr.2023.109217

**Published:** 2023-12-31

**Authors:** Nasrollah Abian, Omid Momen, Fatemeh Esfandiari, Ramin Azarhoush

**Affiliations:** a Department of urology, 5Azar Hospital, School of Medicine, Golestan University of Medical Sciences and Health Services, Gorgan, Iran; b Department of orthopedics, 5Azar Hospital, school of medicine, Golestan University of Medical Sciences and Health Services, Gorgan, Iran; c Department of pathology, 5 Azar Hospital, School of Medicine, Golestan University of Medical Sciences and Health Services, Gorgan, Iran

**Keywords:** Cancer of unknown primary, Renal cell carcinoma, Metastatic RCC, Case report, Sunitinib, Tyrosine kinase inhibitor

## Abstract

**Introduction:**

Although 25–30 % of renal cell carcinomas (RCC) might be diagnosed in metastatic stage, occurrence of metastatic renal cell carcinoma (mRCC) as a cancer of unknown primary site (CUP-mRCC) is extremely rare. Here, we present a case of vertebral mass causing radicular pain that has been diagnosed to be mRCC through core needle biopsy while no renal mass has been found during serial imaging.

**Case presentation:**

A 60-year-old woman presented with severe lumbar pain radiating to left leg. Lumbar X-ray suggested a mass in second lumbar vertebra which was confirmed by MRI. Biopsy showed that the mass was clear cell RCC. Abdominopelvic CT scan and other metastatic work-up found no primary source for the cancer –in kidneys- nor any other metastasis. Tumor resection was performed followed by sunitinib administration. 3 months after the surgery, she is symptom free with no signs of disease progression nor kidney tumor.

**Discussion:**

26 cases of CUP-mRCC has been reported in literature. Lymph nodes are the most commonly involved organ in CUP-mRCC. Exclusive bone involvement –similar to our case- have been reported in only 3 cases. No specific treatment guideline exists but surgery, systemic therapy, combination therapy, and radiotherapy have been used, with the first two items being the most commonly used ones.

**Conclusion:**

Tumor resection plus sunitinib seems to be a reasonable option in solitary CUP-mRCC involving vertebral column. Our patient is symptom free and there are no signs of disease progression nor kidney cancer in follow-up imaging after 3 months of surgery.

## Highlights

• Metastatic RCC may happen in the absence of concomitant renal tumor.
• Multidisciplinary team is needed for a comprehensive evaluation of such disease.
• Surgery plus sunitinib is a reasonable option in CUP-mRCC with solitary bone tumor.

## 1 Introduction

Renal cell carcinoma (RCC) is considered the most lethal cancer in urology. With the advancement in radiological technology, the disease is mostly diagnosed in a local stage while being barely symptomatic, and its classic triad of symptoms i.e., pain, palpable mass, and hematuria rarely does happen. Yet, 25–30 % of RCCs might be diagnosed while metastasis have been occurred [1].

It has been shown that the larger the RCC tumor of kidney is, the higher the risk of metastasis will be; in other words, it is very unlikely to see metastasis in smaller sized kidney RCCs [2]. Much less, is the presence of metastatic RCC (mRCC) in the absence of renal tumor.

26 cases of mRCC as a cancer of unknown primary site (CUP-mRCC) have been reported in English literature; clear cell RCC is the most common type in CUP-mRCC. Lymph nodes are the most common site of involvement in CUP-mRCC [3]. Solitary bone involvement have been scarcer. Here we present a case of vertebral mass causing severe pain and cord compression that has been diagnosed to be RCC by both biopsy and tumor resection while no renal tumor has been found through serial imaging in a 4-month period before and after vertebral tumor surgery.

## 2 Case presentation

A 60-year-old woman presented with severe lumbar pain radiating to flank and left leg for 3 months. She had no urinary symptoms nor gross hematuria. She had no past medical history nor relevant family history. Her physical exam was normal except tenderness on her upper lumbar spines. Her blood cell count, serum biochemistry, and urine analysis were normal.

**Fig. 1:**
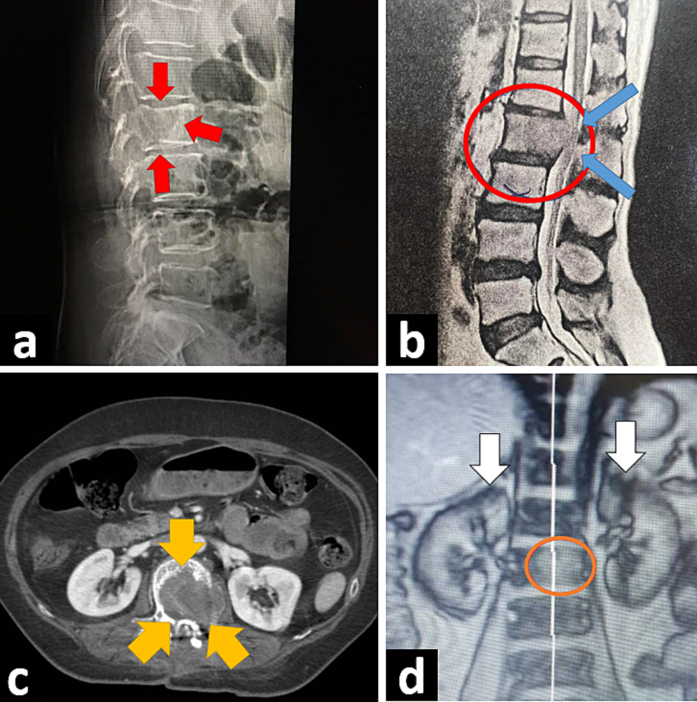
a. Lumbar X-ray (lateral view), showing lytic lesion in second lumbar vertebra (red arrows). b. Lumbar MRI, sagittal view, showing tumor in second lumbar vertebra (red circle) causing cord compression (blue arrows). c. Abdominopelvic CT scan showing vertebral mass (yellow circles) without any kidney tumor. d. Coronal reconstruction of abdominopelvic MRI, showing tumor in vertebra (green circle) and normal kidneys (white arrows). (For interpretation of the references to colour in this figure legend, the reader is referred to the web version of this article.)

Plain lumbar X ray was taken showing a suspected lytic lesion in the body of second lumbar (L2) vertebra (Fig. 1 a). Lumbar MRI revealed 4.5 cm mass in the body and pedicle of L2 expanding posterior cortex into spinal canal (Fig. 1 b). Increased activity in L2 was also seen in bone scan. Core needle biopsy showed tumor cells with clear cytoplasm and oval nuclei. Immunohistochemical (IHC) analysis confirmed positive reactivity for PAX8, EMA, CAIX, CD10, and CK, and negative for p63, CK7, CD34, desmin, S100, Melan-A, SF-1, and AMACR, compatible with the pathologic diagnosis of metastatic clear cell RCC. To find the primary source of tumor, abdominopelvic CT scan with and without IV contrast was taken exhibiting no sign of renal tumor nor lymphadenopathy but the same mass in L2 (Fig. 1 c). In order to complete metastasis workup, chest and brain CT was taken which showed no abnormality. Also, serum lactate dehydrogenase, erythrocyte sedimentation rate, calcium, alkaline phosphatase, and liver function tests were normal.

Having a Karnofsky performance score of 90 % and normal serum calcium levels without any neutrophilia, anemia, or thrombocytosis, she was categorized in intermediate risk group according to International Metastatic Renal Cell Carcinoma Database Consortium (IMDC) risk score as one risk factor was positive: starting systemic therapy within less than a year from diagnosis.

Considering rarity of the disease, a multidisciplinary meeting (MDM) was held with the presence of orthopedist, urologist, hematologic-oncologist, pathologist, and radiologist. Reviewing patient's chief complaint, clinical and laboratory findings, and imaging studies, an abdominopelvic MRI was suggested to be performed as a safety measure and if MRI findings were consistent with previous CT scans, tumor resection would be the primary treatment due to her severe radicular pain, followed by tyrosine kinase inhibitor (TKI) administration.

**Fig. 2:**
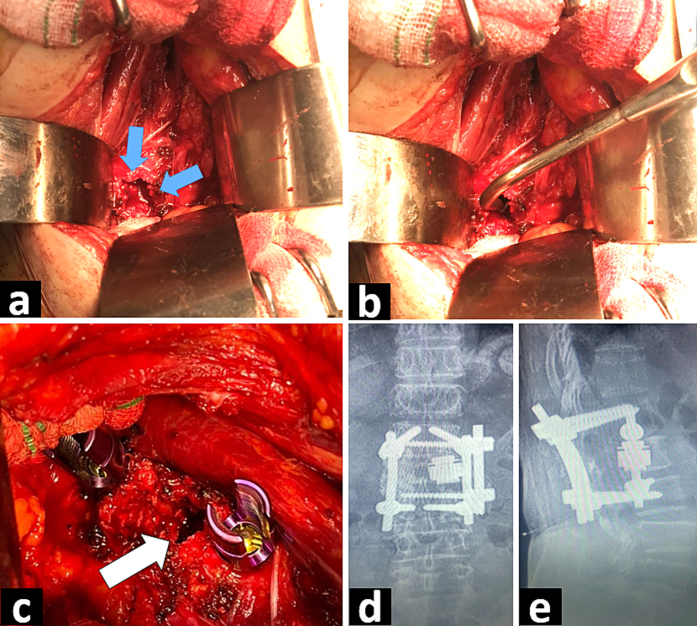
a. L2 vertebral cortex after corpectomy and resection of tumor through flank incision. Blue arrows indicate corpectomy site. b. Yankauer suction placed into resection site of the tumor showing depth of resection. c. Insertion of screw-rod system into first and third lumbar vertebra. White arrow shows site of tumor resection. d. Post-operation lumbar X ray (anteroposterior view), showing mesh cage and the screw-rod system. e. Lateral view of post-operation lumbar X ray. (For interpretation of the references to colour in this figure legend, the reader is referred to the web version of this article.)

**Fig. 3:**
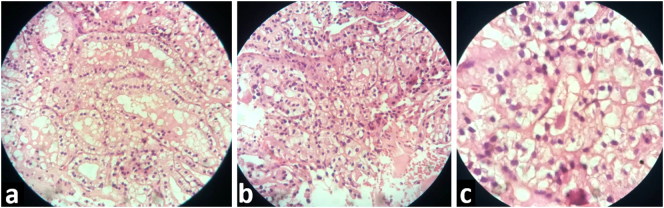
Hematoxylin and eosin stain of the resected mass showing tumor cells with clear cytoplasm and oval nuclei. a. ×40 b. ×100 c. ×400.

**Fig. 4:**
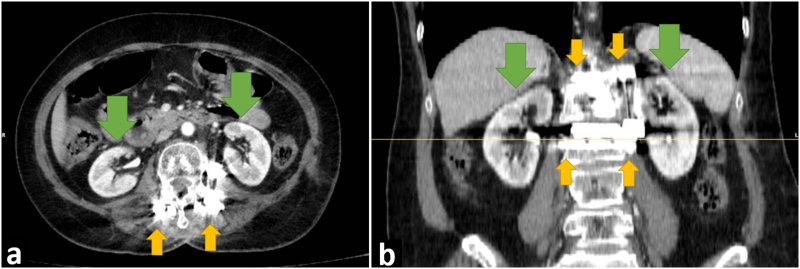
Follow up CT scan 3 months after surgery revealing no tumor progression nor any kidney tumor. Yellow arrows show screw-rod system and green arrows show normal kidneys. a. Transverse section b. Coronal reconstruction of CT scan. Thin yellow line indicates the location of transverse section. (For interpretation of the references to colour in this figure legend, the reader is referred to the web version of this article.)

As abdominopelvic MRI results were similar to the findings of CT scan (Fig. 1 d), corpectomy and pedicle resection along with tumor removal were performed (Fig. 2 a, b). The L2 vertebra was reconstructed by mesh cage filled with allogenic bone and the screw-rod system was inserted into first and third lumbar vertebra through both anterior and posterior approaches (Fig. 2 c, d, e). Pathology was clear cell RCC, same as biopsy (Fig. 3). Sunitinib 50 mg daily (4 weeks on, 2 weeks off) was started after the surgery. Her pain is relieved and she is currently symptom free. Follow up CT scan 3 months after surgery showed no sign of renal tumor or disease progression (Fig. 4). Our case report has been reported in line with the SCARE criteria [4].

## 3 Discussion

As demonstrated by Pierorazio et al., the larger the RCC be, the more invasive its behavior would become [5]. So, presence of metastasis in large kidney RCCs are not surprising; RCC metastasis may rarely be present in smaller renal tumors too [2], but mRCC in the absence of renal tumor is extremely rare. Only 26 cases of CUP-mRCC has been reported in literature. Similar to regular cases of RCC, clear cell RCC is the most common type in CUP-mRCC [3]. On the other hand, the distribution of metastasis originating from kidney RCC tumor differs to metastasis in CUP-mRCC. The most common three sites of metastasis in the presence of kidney tumor are lung, bone, and lymph nodes [6] but in CUP-mRCC, it would be lymph nodes, bones, and adrenal gland, respectively. 6 cases of bone involvement in CUP-mRCC have been reported, half of them had metastatic on other sites as well (lung metastasis in particular) [7,8]. Only 3 cases of CUP-mRCC had exclusive metastasis of bone; in all of them, vertebral column was the site of metastasis [3,7,9], similar to our case.

The mechanism for this phenomenon is not known, but several hypotheses have been proposed in this regard. One of them is spontaneous regression of primary tumor after occurrence of metastasis. Another one is related to the size of tumor: the primary tumors being too small to be found in initial imaging. Thus, serial imaging is advocated which may find the primary tumor in CUP-mRCC. According to third hypothesis, the metastasis might actually be a primary cancer in an ectopic kidney or embryonic mesonephric remnant, same as the embryonic explanation of extragonadal germ cell tumors in relation to testicular cancer [10,11].

Diagnosis is challenging as clear cell tumors may originate from different organs of body. Therefore, IHC is mandatory to reach the most probable diagnosis. Being CK positive highlights presence of epithelial tumor, while negative reactivity for desmin and CD34 dampens possibility of sarcoma. On the other hand, being CD10 positive and p63 negative, lowered the probability of skin or salivary gland origin. Positive reactivity for PAX8, CAIX, EMA, and CD10 combined with negative IHC results for SF-1 (against adrenal origin), S100, and Melan-A (ruling out melanocytic origin) made clear cell RCC our most probable diagnosis [12].

No specific guideline exists on CUP-mRCC management. Biopsy and pathological examination are obviously our main armamentarium in reaching the diagnosis. Yet, as the disease is exceedingly rare, it seems logical to choose the best treatment approach in MDM; reviewing treatment options and choosing the most suitable one for the patient. Most common initial treatments in previous 26 cases were either surgery or systemic therapy; TKIs have been the most common subgroup of systemic therapies used in previous cases. Other treatments included radiotherapy, or combination therapy. While no special conclusion about best treatment option can be inferred from previous cases, metastasectomy -if feasible- seems to be the most logical treatment as the patient with the highest survival period among previous 26 cases had undergone only metastasectomy without systemic therapy [13]. Reported survival period of CUP-mRCC is quite variable ranging from 3 months to more than 60 months [3]. Focusing on CUP-mRCC involving bone only, treatment modality differed in aforementioned 3 cases including immunotherapy [7], radiotherapy [9], and immunotherapy plus palliative radiotherapy [3]. Survival period has been reported from 3 to more than 6 months in these 3 cases [3,7,9].

Our case is the only case of CUP-mRCC exclusively involving bone where surgery was undertaken. While immune checkpoint inhibitors are advocated as the first line therapy in mRCC [14], a more affordable medication covered by insurance companies was chosen so that the patient's drug competence remain high during treatment course. Thus, as she was IMDC intermediate risk, sunitinib was chosen from the alternative medications according to EAU guideline [14] and she is currently under treatment.

The disease showed no progression in the follow up CT scan 3 months after surgery nor any renal mass was found. Our follow-up plan is similar to surveillance program for high-risk patients after partial nephrectomy [15]. Ultrasonography might not be a good option as it cannot show bone tumors and has low sensitivity for small renal masses. MRI might miss tumor presence in organs near inserted orthopedic devices especially the nearby kidneys. Hence, we plan to perform chest and abdominopelvic CT scan every 6 months for the next 3 years and then continue it annually.

## 4 Conclusion

Although CUP-mRCC is extremely rare, it is a possible cause of cancers of unknown primary. Pathological assessment is our main armamentarium in reaching exact diagnosis. Suggested treatment options include surgery, radiotherapy, systemic therapies, or combination of them. As in our case, tumor resection by surgery plus sunitinib is a feasible option in CUP-mRCC involving one vertebra.

## Abbreviations

RCC: Renal cell carcinoma
IHC: Immunohistochemistry
MDM: multidisciplinary meeting
TKI: Tyrosine kinase inhibitor
mRCC: Metastatic RCC
CUP-mRCC: Cancer of unknown primary due to mRCC

## Ethical approval

The ethical approval has been exempted by our institution.

## Funding

This research did not receive any specific grant from funding agencies in the public, commercial, or not-for-profit sectors.

## CRedit authorship contribution statement

Study concept: Omid Momen, Nasrollah Abian.

Data collection: Nasrollah Abian, Omid Momen, Ramin Azarhoush.

Data interpretation: Nasrollah Abian, Fatemeh Esfandiari.

Writing the paper: Nasrollah Abian.

Revision: Omid Momen.

## Guarantor

Nasrollah Abian, Omid Momen.

## Research registration number

Although pretty rare, our case report is not “First in Man”.

## Declaration of competing interest

The authors declare that there is no conflict of interests regarding the publication of this article.
